# Assessing Implementation of Helping Babies Breathe Program Through Observing Immediate Care of Neonates at Time of Delivery

**DOI:** 10.3389/fped.2022.864431

**Published:** 2022-04-25

**Authors:** Martha Mayer, Nomvuyo Xhinti, Vuyiswa Dyavuza, Luzuko Bobotyana, Jeffrey Perlman, Sithembiso Velaphi

**Affiliations:** ^1^Department of Paediatrics, Nelson Mandela Academic Hospital, Walter Sisulu University, Mthatha, South Africa; ^2^Helping Babies Breathe Programme, Resuscitation Council of Southern Africa, Johannesburg, South Africa; ^3^Division of Newborn Medicine, Weil-Cornell Medicine, New York, NY, United States; ^4^Department of Paediatrics, Faculty of Health Sciences, School of Clinical Medicine, Chris Hani Baragwanath Academic Hospital, University of the Witwatersrand, Johannesburg, South Africa

**Keywords:** breathing, neonate, training, helping babies breathe, umbilical cord

## Abstract

**Background:**

Training in neonatal resuscitation has been shown to reduce deaths related to intrapartum asphyxia. Helping Babies Breathe (HBB) is a simulation-based program focusing on training healthcare providers (HCPs) in immediate neonatal care including stimulation, initiating bag mask ventilation (BMV) in the absence of breathing by 1 min of life, and delayed (30–60 s after birth) umbilical cord clamping (DCC). Data on implementation of HBB posttraining are limited.

**Objective:**

To determine time from birth to spontaneous breathing, cord clamping, and initiation of BMV in a setting where the majority of HCPs are HBB trained.

**Methods:**

Two research nurses observed deliveries conducted in two referral hospitals. Timing included the onset of breathing, cord clamping, and initiation of BMV. Deliveries were grouped according to the mode of delivery.

**Results:**

In total, 496 neonates were observed; 410 (82.7%) neonates cried or had spontaneous breathing (median time 17 s) soon after birth, 25/86 (29%) of neonates not breathing responded to stimulation, 61 (12.3%) neonates required BMV, and 2 (0.4%) neonates required chest compression and/or adrenalin. Neonates delivered by cesarean section (CS) took longer to initiate first breath than those delivered vaginally (median time 19 vs. 14 s; *p* = 0.009). Complete data were available in 58/61 (95%) neonates receiving BMV, which was initiated in 54/58 (93%) cases within 60 s of life (the “Golden Minute”). Median time to cord clamping was 74 s, with 414 (83.5%) and 313 (63.0%) having cord clamped at ≥ 30 and ≥ 60 s, respectively. Factors associated with BMV were CS delivery [odds ratio (OR) 29.9; 95% CI 3.37–229], low birth weight (LBW) (birthweight < 2,500 g) (OR 2.47; 95% CI 1.93–5.91), and 1 min Apgar score < 7 (OR 149; 95% CI 49.3–5,021). DCC (≥ 60 s) was less likely following CS delivery (OR 0.14; 95% CI 0.02–0.99) and being LBW (OR 0.43; 95% CI 0.24–0.77).

**Conclusion:**

Approximately 83% of neonates initiated spontaneous breathing soon after birth and 29% of neonates not breathing responded to physical stimulation. BMV was initiated within the Golden Minute in most neonates, but under two-thirds had DCC (≥60 s). HBB implementation followed guidelines, suggesting that knowledge and skills taught from HBB are retained and applied by HCP.

## Introduction

Approximately 85% of newborns breathe spontaneously at birth and another 5–10% will start breathing with just physical stimulation (drying and rubbing) (1–3). This implies that most newborns will do well with routine care of drying them and maintaining warmth. Conversely, less than 10% of deliveries will require some assistance with breathing. The recommended form of assisting any infant with poor respiratory effort is through providing positive pressure ventilation using a bag mask resuscitator. If bag mask ventilation (BMV) is started promptly, most babies will respond and initiate breathing immediately. In those who do not breath despite 30–60 s of BMV, the heart rate is assessed and chest compressions are recommended if the heart rate is less than 60 beats per minute (bpm) and adrenalin if the heart rate is < 60 bpm despite 30–60 s of chest compressions (4). It is reported that of the total deliveries, less than 1% of patients will need chest compressions and/or adrenalin (1, 2, 5, 6).

At the time of birth, the umbilical cord is clamped thereby removing the placental circulation. The clamping of the cord is considered to be delayed, if it occurs at or more than 30–60 s (7) from the time of delivery of the baby, using ≥ 30 s for preterm and term (8, 9) and some recommended ≥ 60 s for term infants (10, 11). A meta-analysis reported that delayed clamping of the cord is associated with a number of benefits including less incidence of intraventricular hemorrhage, less incidence of necrotizing enterocolitis, and less need for blood transfusion in preterm infants (12); while in term infants, it is associated with less incidence of iron deficiency anemia at 6 months of age (13). The International Liaison Committee on Resuscitation (ILCOR) and the American Heart Association Guidelines for Cardiopulmonary Resuscitation and Emergency Cardiovascular Care recommend to delay cord clamping for at least 30 s in newborns who do not require resuscitation at birth (14, 15).

The Helping Babies Breathe (HBB) program is a simulation-based training program. It trains the healthcare providers (HCPs) on the resuscitation of the neonate focusing on ensuring that babies who are not breathing are assisted with BMV by the first minute of life. The steps of resuscitation in the HBB program include suctioning if there is meconium, drying the infant, checking if the baby is crying or breathing, delaying clamping of the cord, keeping the baby warm, and starting BMV, if the baby is not breathing within the first minute of life (Golden Minute) (16, 17). The implementation of this training program has been associated with a reduction in early neonatal mortality rates (ENMRs) and fresh stillbirth rate (FSBR) (18, 19).

Several hospitals in the Eastern Cape, South Africa, escalated training of their HCP on immediate care of newborns at birth using HBB from 2016. But for any program to be successful, it needs proper implementation. Training on a certain skill using simulation requires that a follow-up is made to assess whether the individual is applying the skill appropriately in a real-life situation. The objective of this study was to assess the condition of newborn infants at birth and the implementation of skills or procedures recommended in the HBB training program, in a setting where the majority of HCP were trained in this program.

## Patients and Methods

### Study Design and Setting

This was a prospective, observational study. It was conducted at two referral hospitals adjacent to one another in the city of Mthatha, Eastern Cape, South Africa. These hospitals are Mthatha Regional Hospital (MRH) and Nelson Mandela Academic Hospital (NMAH), a tertiary public hospital. Both the hospitals are referral centers for the clinics, district, and regional hospitals in the Oliver Reginald (OR) Tambo region, Eastern Cape, South Africa. MRH and NMAH conduct about 6,000 and 4,000 deliveries per annum, respectively. The majority of vaginal deliveries are conducted by midwives, with doctors mainly delivering those who require cesarean section.

### Study Population and Procedure

Deliveries conducted at the two hospitals during weekdays, daytime (08:00–16:00 h) from January to December 2017, were observed. Two research nurses observed deliveries and HBB-related activities conducted by HCP at the time and soon after delivery. Maternal characteristics, pregnancy, and labor details of deliveries observed were recorded. The research nurses timed the onset of breathing and clamping of the cord from the time of complete delivery of the body using a stopwatch. Interventions (stimulation and initiation of BMV) and time of their implementation among infants who were not breathing spontaneously at birth were also observed and recorded. Delayed cord clamping was defined as clamping the cord at 30–60 s or longer to cater for both the preterm and term infants.

Time to spontaneous breathing was categorized in 10 s epochs from 0 to 60 s. Comparisons were made between those who were delivered vaginally and those by cesarean section in terms of time to first cry, breath, and clamping of the cord. Factors associated with the need for BMV and for delayed clamping of the cord (for both ≥ 30 and ≥ 60 s definition) were assessed using the univariate and multiple logistic regression reported as odds ratios and 95% CI. Only variables with *p*-values < 0.05 were included in the multivariate analysis. Difference with *p*-values < 0.05 was considered as statistically significant. This study was conducted after getting approval from the Human Research Ethics Committee of the Walter Sisulu University.

## Results

### Characteristics of Observed Deliveries

A total of 496 deliveries were observed. Maternal (pregnancies and labor events) and infants’ characteristics of deliveries observed are shown in Table 1. The majority (71.6%) of mothers delivered were of ages 20–35 years and 14.5% were teenagers (<20 years). Almost all the mothers (99.0%) attended the antenatal clinic, 34.1% were primigravida, and 48.4% were delivered preterm (<37 weeks). Hypertensive disorders (21.3%), previous cesarean section (13.3%), fetal distress (8.6%), and cephalopelvic disproportion (6.0%) were common pregnancy or labor-related complications in the deliveries observed. Two-thirds (66.7%) were delivered by cesarean section and 68.2% of all the deliveries were conducted by medical doctors. The infants’ average birth weight and gestational ages were 2,928 ± 672 g and 36 ± 2 weeks, respectively, with 103 (20.7%) being of low birth weight (birth weight < 2,500 g).

**TABLE 1:** Maternal and infant characteristics.

Characteristics	Number	Percent
**Maternal characteristics**		
Maternal age		
<20 years	72	14.5
20–35 years	355	71.4
>35 years	70	14.1
**Gravida**		
<2	169	34
2–4	292	58.8
>4	36	7.2
HIV positive	172	34.8
Cephalic presentation	477	96
Preterm (<37 weeks)	241	48.5
Delivered by cesarean section	332	66.8
Delivery attended by midwife	158	31.8
**Complications during pregnancy/labor**		
None	164	33
Hypertensive disorders	106	21.3
Previous cesarean section	66	13.3
Fetal distress	44	8.9
Cephalopelvic disproportion	30	6
Poor progress in labor	17	3.4
Maternal illness other than hypertension	16	3.2
Multiple pregnancy	12	2.4
Others	42	8.5
**Infant characteristics**		
Male sex	250	50.4
**Apgar score at 1 min**		
Number with Apgar < 7	33	6.7
Number with Apgar score 7–10	463	93.3
**Apgar score at 5 min**		
Number with Apgar < 7	6	1.2
Number with Apgar score 7–10	490	98.8
**Birth weight (in grams)**		
Mean (SD)	2,928 (672)	_
Median (IQR)	3,000 (2,565–3,400)	_
Range	750–5,030	_
Number with weight < 2,500 g	103	20.8
**Gestational age (in weeks)**		
Mean (SD)	36 (2)	_
Median (IQR)	37 (36–38)	_
Range	26–42	_
Number with gestation < 37 weeks	240	48.4
Total deliveries observed	496	100

*SD, Standard deviation; IQR, Interquartile range.*

### Need for Stimulation and Bag Mask Ventilation

**FIGURE 1:**
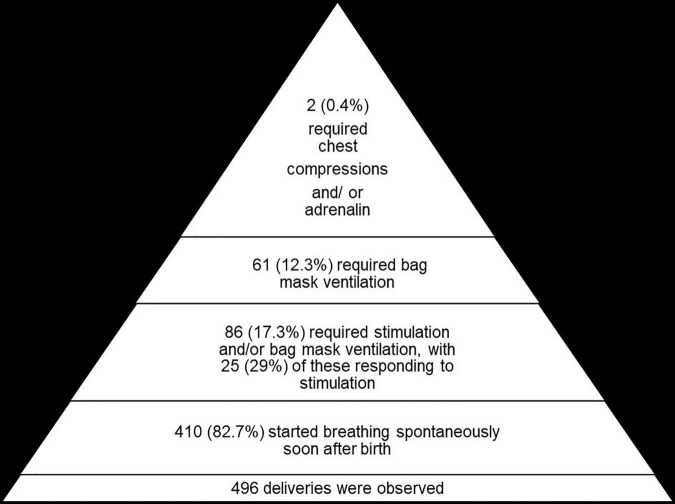
The number of deliveries and proportions requiring stimulation, bag mask ventilation, and chest compressions.

In total, 410 (82.7%) infants started breathing spontaneously soon after birth. Among the 86 (17.3%) not breathing spontaneously, 25 (29.1%) started breathing after physical stimulation, leaving 61 (12.7%) requiring BMV (Figure 1). Among the 61 who required BMV, 58 (95.1%) had time for initiating BMV recorded and in 54 (93.1%) of these, BMV was initiated by 1 min of life. Two (0.4%) neonates required chest compressions and/or adrenalin.

### Time to First Spontaneous Breath and Time to First Cry

**FIGURE 2:**
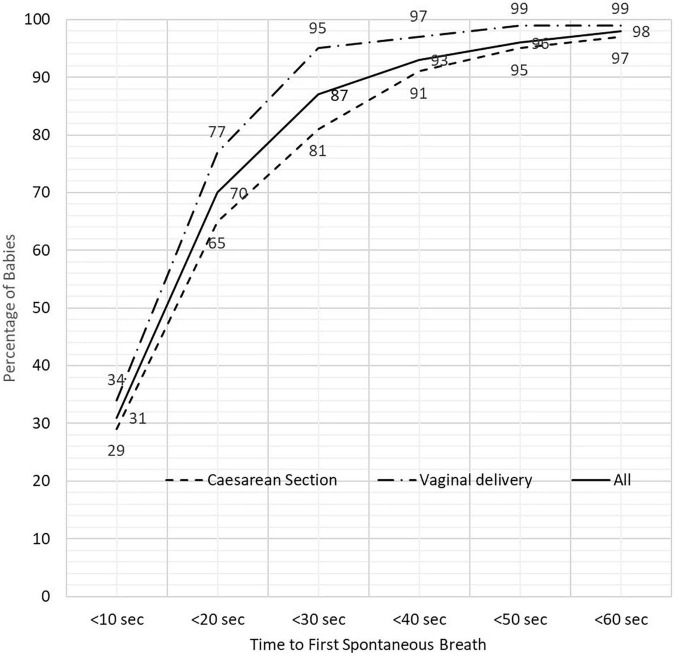
The proportion of newborn babies breathing spontaneously according to time after birth and mode delivery.

Overall, 87% of neonates had taken their first breath by 30 s. Looking at different time points, there was a lower proportion who had achieved spontaneous breathing among those born by cesarean section compared to vaginal deliveries, with 81% of those delivered by cesarean section achieving spontaneous breathing at 30 s compared to 95% of those born vaginally (*p* < 0.001) (Figure 2). At 1 min (60 s), 98% of neonates had initiated breathing either spontaneously or after stimulation with or without BMV and the remainder who were not breathing had BMV started after 60 s.

The median time from birth to first spontaneous breath and crying was 17 and 29 s, respectively. Neonates delivered by cesarean section took longer to initiate their first breath or start crying compared to those who were delivered vaginally (median time 19 vs. 14 s to first breath; *p* = 0.009 and 31.5 vs. 28 s to start crying, *p* < 0.001) (Table 2). The median time for initiation of first breath was not statistically different between term and preterm infants (median time 16 vs. 18 s, *p* = 0.376). Infants born by cesarean vs. vaginal took longer to first breath even after grouping babies according to gestational age (term vs. preterm) with median time for those following cesarean section 17 vs. 13 s (*p* = 0.004) in term and 22 vs. 13.5 s (*p* < 0.001) in preterm infants. On the univariate analysis, neonates requiring BMV were more likely to have been born by cesarean section (OR 6.49; 95% CI 2.50–16.9), being born preterm (37 weeks) (OR 2.23; 95% CI 1.26–3.92), be of low birth weight (<2,500 g) (OR 4.73; 95% CI 2.65–8.47), and to have Apgar scores < 7 at 1 min (OR 149.1; 95% CI 29.0–767.6) and 5 min (OR 47.0; 95% 5.16–436.3) compared to those who started breathing spontaneously or responded to stimulation. On the multivariate analysis, only neonates who were born by cesarean section, of low birth weight, and had an Apgar score < 7 were associated with the need for BMV (Table 3).

**TABLE 2:** Time to first breath, first cry, and clamping of the cord from birth according to the mode of delivery in term and preterm infants.

	All neonates (*N* = 496)			
Event observed	All	Vaginal delivery *N* = 165	Cesarean section *N* = 331	Comparing vaginal and cesarean section deliveries *p*-value
Time to first breath (s)	17 (9–29)*	14 (8–21)	19 (10–38)	0.009
Time to first cry (s)	29 (17–47)	28 (17–38)	31.5 (15–55.5)	<0.001
Time to Cord Clamping (s)	74 (39–117)	139 (112–153)	58 (30–82.5)	<0.001

	**Term (gestation ≥ 37) neonates (*N* = 256)**			

	**All**	**Vaginal delivery *N* = 81**	**Cesarean section *N* = 175**	

Time to first breath (s)	16 (9.5–29)	13 (9–21)	17 (10–34)	0.004
Time to first cry (s)	28 (17–43)	28 (17–36)	29 (17–50)	0.13
Time to cord clamping (s)	76.5 (38.5–113.5)	141 (112–153)	58 (30–84)	<0.001

	**Preterm (gestation < 37 weeks) neonates (*N* = 240)**			

	**All**	**Vaginal delivery *N* = 84**	**Cesarean section *N* = 156**	

Time to first breath (s)	18 (9–32)	13.5 (8–20.5)	22 (10–44)	<0.001
Time to first cry (s)	31 (17–53)	27.5 (17–39)	34.5 (17.5–63.5)	0.024
Time to cord clamping (s)	74 (42–118)	137 (105–152)	57.5 (31–78.5)	<0.001

*Median (interquartile range).

**TABLE 3:** Comparing neonates requiring bag mask ventilation and those who did not.

Characteristics	Breathing spontaneously and/or responded to stimulation	Needed bag mask ventilation	Comparing those breathing spontaneously and need for bag mask ventilation			
	*N* = 435	*N* = 61	Univariate analysis odds ratio (95% CI)	*p*-value	Multivariate analysis odds ratio (95% CI)	*p*-value
**Maternal age**						
<20 years	61 (14.0)	11 (18.0)	Ref	-	N/A	
20–35 years	315 (72.4)	40 (65.6)	0.07 (0.34–1.45)	0.339	-	-
>35 years	59 (13.6)	10 (16.4)	0.92 (0.36–2.35)	0.868	-	-
**Gravidity**					N/A	
<2	144 (33.1)	25 (41.0)	Ref	-		
2–4	264 (60.7)	28 (45.9)	0.61 (0.34–1.09)	0.092	-	-
>4	27 (6.2)	8 (13.1)	1.65 (0.67–4.04)	0.272	-	-
**Attended antenatal care**					N/A	
No	4 (0.9)	1 (1.6)	Ref	-	-	-
Yes	431 (99.1)	60 (98.4)	0.56 (0.06–5.07)	0.597	-	-
**HIV positive**					N/A	
No	279 (64.5)	43 (70.5)	Ref	-	-	-
Yes	154 (35.5)	18 (29.5)	0.76 (0.42–1.37)	0.359	-	-
**Mode of delivery**						
Vaginal	160 (36.7)	5 (8.2)	Ref	-	Ref	-
Cesarean section	275 (63.3)	56 (91.8)	6.49 (2.50–16.9)	<0.001	29.9 (3.37–229)	0.002
**Presenting part at birth**						
Non-cephalic	12 (2.8)	7 (11.5)	Ref	-	Ref	-
Cephalic	423 (97.2)	54 (88.5)	0.24 (0.09–0.63)	0.002	0.62 (0.15–2.57)	0.509
**Gestational age**						
≥37 weeks	235 (54.0)	21 (34.4)	Ref	-	Ref	-
<37 weeks	200 (46.0)	40 (65.6)	2.23 (1.26–3.92)	0.004	1.06 (0.45–2.51)	0.885
**Birth weight**						
≥2,500 grams	362 (83.2)	31 (50.8)	Ref	-	Ref	-
<2,500 grams	73 (16.8)	30 (49.2)	4.73 (2.65–8.47)	<0.001	2.47 (1.03–5.91)	0.043
**Sex**					N/A	
Female	217 (49.8)	29 (47.5)	Ref	-	-	-
Male	218 (50.1)	32 (52.5)	1.09 (0.64–1.87)	0.774	-	-
**Apgar score at 1 min**						
7–10	433 (99.5)	30 (49.2)	Ref	-	Ref	-
< 7	2 (0.5)	31 (50.8)	149.1 (29.0–767.6)	<0.001	497 (49–5,021)	<0.001
**Apgar score at 5 min**						
7–10	435 (100)	55 (90.2)	Ref	-	Ref	-
<7	0	6 (9.8)	Infinite	<0.001	0.46 (0.02–10.8)	0.632

### Cord Clamping

The proportion who had clamping of the cord delayed was 83.4% (414/496) using a definition of delayed cord clamping as ≥ 30 s and 63.1% (313/496) using the definition of ≥ 60 s. There were no differences in the proportion who had delayed cord clamping between term and preterm infants for both ≥ 30 s (83.2 vs. 83.8%) and ≥60 s (63.7 vs. 62.5%). The umbilical cord was clamped at a median time of 74 s, with neonates delivered by cesarean section having their umbilical cords clamped earlier than those delivered vaginally (median 58 vs. 139 s; *p* < 0.001). This difference was observed for both the preterm and term infants (Table 2).

In using the definition of delayed cord clamping as ≥ 30 s, the median time to first breath was not different between those with early (<30 s) and delayed cord clamping (median time 16 vs. 17 s, *p* = *0.204*) (Table 4). On the multivariate analysis, the only factor associated with lower odds (OR 0.10, 95% CI 0.01–0.91, *p* = *0.041*) of having delayed cord clamping was an infant being delivered by cesarean section.

**TABLE 4:** Comparing characteristics of deliveries with early (<30 s) vs. delayed (≥30 s) cord clamping.

	Early cord clamping	Delayed cord clamping	Comparing early and delayed cord clamping			
Characteristics	*N* = 82	*N* = 414	Univariate Analysis Odds ratio (95% CI)	*p*-value	Multivariate analysis Odds ratio (95% CI)	*p*-value
**Mode of delivery**						
Vaginal	4 (4.9)	161 (39.9)	Ref	-	Ref	
Cesarean	78 (95.1)	253 (61.1)	0.08 (0.03–0.23)	<0.001	0.10 (0.01–0.91)	0.041
**Healthcare worker**						
Doctor	78 (95.1)	260 (62.8)	Ref	-	Ref	-
Nurse	4 (4.9)	154 (37.2)	11.6 (4.0–33.4)	<0.001	1.27 (0.15–11.1)	0.831
**Gestational age**					N/A	
≥37 weeks	43 (52.4)	213 (51.5)	Ref	-	-	
<37 weeks	39 (47.6)	201 (48.6)	1.04 (0.65–1.67)	0.87	-	
**Birth weight**						
≥2,500 g	57 (69.5)	336 (81.2)	Ref	-	Ref	-
<2,500 g	25 (30.5)	78 (18.8)	0.52 (0.31–0.90)	0.018	0.76 (0.43–1.35)	0.351
**Apgar score at 1 min**						
7–10	71 (86.6)	392 (94.7)	Ref	-	Ref	-
<7	11 (13.4)	22 (5.3)	0.36 (0.17–0.78)	<0.001	0.70 (0.22–2.26)	0.547
**Apgar score at 5 min**						
7–10	79 (96.3)	411 (99.3)	Ref	-	Ref	-
<7	3 (3.7)	3 (0.7)	0.19 (0.04–0.98)	0.026	0.27 (0.03–2.14)	0.215
**Bag mask ventilation**						
No	63 (76.8)	372 (89.9)	Ref	-	Ref	-
Yes	19 (23.2)	42 (10.1)	0.37 (0.20–0.69)	<0.001	0.80 (0.34–1.91)	0.618
**Time to first breath (s)**						
All	16 (9–48)	17 (9–28)	N/A	0.204	N/A	N/A
Vaginal	70 (41.5–73.5)	13 (8–20)	N/A	0.006	N/A	N/A
Cesarean section	16 (8–40)	19 (11–38	N/A	0.592	N/A	N/A

In defining delayed cord clamping as ≥ 60 s, the median time to first breath was shorter for those with delayed cord clamping with median time 30 vs. 14 s, *p* < 0.001 (Table 5).

**TABLE 5:** Comparing characteristics of deliveries with early (<60 s) vs. delayed (≥60 s) cord clamping.

	Early cord clamping	Delayed cord clamping	Comparing early and delayed cord clamping			
Characteristics	*N* = 183	*N* = 313	Univariate analysis Odds ratio (95% CI)	*p*-value	Multivariate analysis Odds ratio (95% CI)	*p*-value
**Mode of delivery**						
Vaginal	12 (6.6)	153 (48.9)	Ref	-	Ref	
Cesarean	171 (93.4)	160 (51.1)	0.07 (0.04–0.15)	<0.001	0.14 (0.02–0.99)	0.048
**Healthcare worker**						
Doctor	173 (94.5)	165 (52.7)	Ref	-	Ref	-
Nurse	10 (5.5)	148 (47.3)	15.6 (7.37–33.0)	<0.001	3.23 (0.45–23.4)	0.246
**Gestational age**					N/A	
≥37 weeks	93 (50.8)	163 (52.1)	Ref	-	-	_
<37 weeks	90 (49.2)	150 (47.9)	0.94 (0.65–1.35)	0.741	-	-
**Birth weight**						
≥2,500 g	119 (65.0)	274 (87.5)	Ref	-	Ref	-
<2,500 g	64 (35.0)	39 (12.5)	0.26 (0.16–0.42)	<0.001	0.43 (0.24–0.77)	0.005
**Apgar score at 1 min**						
7-10	150 (82.0)	313 (100)	Ref	-	Ref	-
<7	33 (18.0)	0	Infinite	<0.001	Infinite	<001
**Apgar score at 5 min**						
7-10	177 (96.7)	313 (100)	Ref	-	Ref	-
<7	6 (3.3)	0	Infinite	<0.001	Infinite	<0.001
**Bag mask ventilation**						
No	122 (66.7)	313 (100)	Ref	-	Ref	-
Yes	61 (33.3)	0	Infinite	<0.001	Infinite	<0.001
**Time to first breath (s)**						
All	30 (12–56)	14 (8–22)	N/A	<0.001	N/A	N/A
Vaginal	43.5 (11–59)	13 (8–19)	N/A	0.008	N/A	N/A
Cesarean section	30 (12–56)	16 (8.5–25)	N/A	<0.001	N/A	N/A

On the multivariate analysis, factors associated with lower odds of having delayed cord clamping were being born by cesarean section (OR 0.14, 95% CI 0.02–0.99), having low birth weight (OR 0.43, 95% CI 0.24–0.77), and Apgar score lower than 7 at 1 min and 5 min and need for BMV.

## Discussion

The main findings in this study are that 83% of neonates started breathing spontaneously soon after birth and this increased to 87% with physical stimulation. Approximately a third (29%) of neonates who were not breathing at birth started breathing spontaneously after physical stimulation. Less than 1% required advanced resuscitation (chest compressions and/or adrenalin). The median time for neonates to start breathing spontaneously from the time of birth was 17 s. Deliveries conducted in this setting where HCPs were trained in the HBB program resulted in 98% of neonates breathing spontaneously or receiving BMV by 1 min of life. Neonates who were born by cesarean section and those with low birth weight were more likely to require BMV and, therefore, were less likely to have delayed cord clamping.

The time to onset of spontaneous respiration in this report was longer than the 10 s observed in a study by Ersdal et al. (20). The proportion of neonates who required BMV in this study is higher than that of 3–6% reported in a meta-analysis looking at the annual number of all the newborns requiring assistance to breath (1, 2), but similar to that reported in a study conducted in Tanzania (20, 21). The possible reason for the longer time to the initiation of spontaneous respiration and a high proportion of babies requiring respiratory support is that the centers where this study was conducted are referral centers and, therefore, are more likely to be delivering high-risk pregnancies. This is supported by the high cesarean section and preterm rates of 66.8 and 48.4%, respectively, in this study. Preterm births and emergency cesarean section have been associated with the need for positive pressure ventilation at birth (22). The proportion of cesarean sections that were performed as emergencies was not recorded in this study. The high proportion of a need for assistance with breathing highlights the importance of having a person with knowledge and skill in basic neonatal resuscitation as taught in HBB, especially for deliveries from high-risk pregnancies.

Though not all the neonates who were not breathing by 1 min received BMV, it was encouraging that 98% of babies were breathing or being ventilated by the “Golden Minute.” This suggests that HBB training enhanced the knowledge and skills of healthcare workers on immediate care of newborn babies and that the goal of making sure that each neonate should be breathing or being assisted to breathe by 1 min was achieved in most deliveries. An association between retention of skills from the HBB training and a positive result in terms of starting BMV within the Golden Minute for those newborns who fail to initiate spontaneous breathing has been reported previously (23). The American Heart Association recommends that each delivery must be attended by a HCP who is skilled, whose sole responsibility is to look after the newborn at birth, so that there are no delays in initiation of resuscitation (15). Delays in initiating positive pressure ventilation are associated with an increase in mortality, with delays of 30 s increasing the risk of mortality or prolonged hospitalization by 12–16% (3, 21).

The ILCOR guidelines suggest that cord clamping should be delayed in neonates who do not require resuscitation (14, 24). The WHO also recommends delayed cord clamping, with the cord being clamped after 1 min–3 min after birth (25). The HBB program recommends that in babies who are crying and/or breathing spontaneously at birth, clamping of the cord should be delayed (16, 17). Overall, approximately two-thirds of neonates had cord clamped at 60 s or longer, with the median time to cord clamping of 74 s, while more than 80% had delayed cord clamping if one uses 30 s or longer to define delay. Cesarean section and infants with low birth weight were found to be less likely to have delayed cord clamping. These factors were also found to be associated with the need for BMV and under such circumstances, the cord was clamped earlier because the HCP wanted to initiate resuscitation and avoid delays. It has been reported previously that neonates who require resuscitation at birth have their cords clamped earlier (20). On the univariate analysis, midwives were more likely to be the ones who performed delayed cord clamping than doctors, supporting that training of midwives in HBB is critical in improving the quality of immediate care of neonates at birth. The most likely reason for doctors not performing as midwives is that they tend to rotate between departments; therefore, some of them might have forgotten what they were trained during HBB. The better performance of midwives in implementing delayed cord clamping compared to doctors has been observed before (26). After adjusting for cesarean section deliveries, the advantage of midwives in performing cord clamping was lost, suggesting that this intervention was not performed very well by both the categories of HCP, even if midwives did better than doctors. In order to improve on delaying cord clamping in neonates who need resuscitation, the setting at the delivery room or operating theater will need to be reorganized to allow initiating BMV while cord clamping is delayed. In developed countries, bed designs that allow initiating resuscitation at the mothers bedside are being explored (27).

The strength of this study is that the settings in which the study was conducted managed high-risk pregnancies and, therefore, presented HCPs with opportunities to implement all the steps taught in the HBB program, as they got exposed to different clinical scenarios. This allowed the study to have a high number of events where HCPs could be assessed. The limitations of the study are that not all the deliveries were observed, as there were limited resources to provide research nurses to observe deliveries at night and during the weekends. Secondly, there is no data on the performance of the interventions taught in HBB before the implementation of this training in order to assess the impact of training on HBB in performing these interventions.

## Conclusion

In a setting where training in the immediate care of the newborn was implemented using the HBB program, HCPs implemented the interventions in immediate care according to the steps of the HBB training program. This highlights that knowledge and skills taught during HBB were retained by most HCPs. More than 10% of neonates required some form of assistance soon after birth highlighting the importance of training in HBB, since not providing the necessary basic interventions such as physical stimulation and BMV may have resulted in the deaths of these neonates. It is known that training in basic neonatal resuscitation can reduce neonatal mortality by a third; therefore, the HBB program should be implemented globally especially in low- and middle-income countries where the rate of intrapartum deaths is high.

## Data Availability Statement

The raw data supporting the conclusions of this article will be made available by the authors, without undue reservation on request.

## Ethics Statement

This study was reviewed by the Walter Sisulu University Human Ethics Research Committee. Written informed consent for participation was not required for this study in accordance with the national legislation and the institutional requirements instead verbal consent was obtained.

## Author Contributions

All authors listed have made a substantial, direct, and intellectual contribution to the work, and approved it for publication.

## Conflict of Interest

The authors declare that the research was conducted in the absence of any commercial or financial relationships that could be construed as a potential conflict of interest.

## Publisher’s Note

All claims expressed in this article are solely those of the authors and do not necessarily represent those of their affiliated organizations, or those of the publisher, the editors and the reviewers. Any product that may be evaluated in this article, or claim that may be made by its manufacturer, is not guaranteed or endorsed by the publisher.
